# Can Yerba Mate Reduce the Risk of Cognitive Impairment? A pilot study

**DOI:** 10.1002/alz70860_100582

**Published:** 2025-12-23

**Authors:** Afra Gilbert, Emilia Gatto, Nicolas Chocobar, Josefina Seguí, Beatriz Crotti, Jonathan Cubas Guillen, Xavier Merchan del Hierro, Lucia Bacigalupe, Gabriel Gustavo Persi, Carlos Mario Melcon, Santiago O'Neill, Claudia Patricia Múnera Martínez, Natalia Sierra Sanjurjo, Galeno Rojas, Liliana Teresita Tribbia, Irene Taravini

**Affiliations:** ^1^ Sanatorio de la Trinidad Mitre, Buenos Aires, Argentina; ^2^ Sanatorio de la Trinidad Mitre, City of Buenos Aires, Argentina; ^3^ Sanatorio de la Trinidad Mitre, City of Buenos Aires, Argentina; ^4^ Departmento de Neurología Cognitiva y Neuropsicología Fleni, C.A.B.A, Buenos Aires, Argentina; ^5^ Ramos Mejía Hospital, Buenos Aires, Buenos Aires, Argentina; ^6^ Fundación para la investigación en Neuroepidemiología, City of Buenos Aires, Argentina; ^7^ Neuroscience Institute, Favaloro Foundation, Buenos Aires, Argentina; ^8^ Neuroscience Institute Favaloro Foundation, Ciudad Autónoma de Buenos Aires, Argentina; ^9^ Instituto de neurociencias Fundacion Favaloro, Buenos Aires, Buenos Aires, Argentina; ^10^ Institute of Neuroscience Favaloro Foundation, Buenos Aires, Argentina; ^11^ Universidad Nacional de Entre Rios, Entre Rios, Argentina; ^12^ Laboratorio de Neurobiología Experimental. ICTAER‐FBRO‐UNER, Entre Rios, Argentina

## Abstract

**Background:**

Yerba mate (YM)**,** an infusion made from *Ilex paraguariensis*, is widely consumed in Argentina and other South American countries. Experimental studies have shown that YM enhances antioxidant defenses, increases chaperonin expression, and reduces AChE activity and Aβ expression. Our objective is to evaluate the potential association between YM consumption and the risk of developing cognitive impairment (CI).

**Method:**

This study was conducted at the Trinidad Mitre Hospital and Favaloro Foundation in Buenos Aires, Argentina, from January 2023 to December 2024. A structured survey was administered to individuals aged ≥50 years who underwent neurocognitive testing. Demographic, epidemiological, and lifestyle data were collected, focusing on YM consumption habits. The control group consisted of individuals with Clinical Dementia Rating (CDR) 0, while the case group included those with CDR ≥0.5 and amnestic profile. Logistic regression was used to analyze the association between the outcome and exposure, adjusted for age and gender.

**Result:**

39 controls‐**C‐**, mean age 68.9 ±8.5 y.o. and 43 patients‐ **P**‐, mean age 73.2± 9.87 y.o. were included, (*p* = NS). The mean years of education are similar in both groups **C** 14.8±3.24 vs. **P**: 14.1 ±3.54. This suggest a possible association between regular consumption of YM and a lower risk of CI (OR 0.76, 95% CI 0.20 ‐ 2.73). Regular YM consumers showed better cognitive performance in logical memory (immediate and delayed recall) and serial word learning.

**Conclusion:**

This pilot study suggests a possible association between YM consumption and reduced CI risk, consistent with previous research. Larger studies are needed to validate this association and explore underlying mechanisms.

